# Choroidal change in acute anterior uveitis associated with human leukocyte antigen-B27

**DOI:** 10.1371/journal.pone.0180109

**Published:** 2017-06-28

**Authors:** Seong Joon Ahn, Ji Hong Kim, Byung Ro Lee

**Affiliations:** Department of Ophthalmology, Hanyang University Hospital, Seoul, Republic of Korea; Oregon Health and Science University, UNITED STATES

## Abstract

**Purpose:**

To evaluate choroidal changes in eyes with acute anterior uveitis associated with human leukocyte antigen (HLA)-B27

**Methods:**

In 44 patients with first-onset, unilateral, acute-onset (<1 week) anterior uveitis for which diagnostic work-ups revealed positivity only for HLA-B27, wide-field three-dimensional volumetric raster scan using swept-source optical coherence tomography was performed for both eyes. Choroidal thickness was measured by automated segmentation and thickness mapping and compared between eyes with uveitis and the fellow eyes at baseline. Choroidal thickness was compared before and after topical and/or systemic corticosteroid therapy. Relative choroidal thickening was defined as the choroidal thickness of the uveitic eye minus that of the corresponding eye and correlated with the degree of intraocular inflammation.

**Results:**

Compared to the fellow eyes, eyes with acute anterior uveitis showed significant choroidal thickening on the subfoveal and parafoveal areas at baseline (all P <0.05). En face choroidal imaging showed dilation of large choroidal vessels on the macula. Relative choroidal thickening significantly correlated with the degree of anterior chamber inflammation at baseline (correlation coefficient = 0.341, P = 0.023). After treating inflammation, the choroid on the macula thinned significantly (from 262.1 ± 66.5 to 239.5 ± 61.0 μm in the subfoveal choroid, P<0.001).

**Conclusions:**

Eyes with HLA-B27-associated anterior uveitis showed significant choroidal thickening at acute phase that subsequently decreased after treatment, indicating subclinical choroidal inflammation in the eyes. Choroidal thickness might indicate disease activity in acute anterior uveitis associated with HLA-B27.

## Introduction

Anterior uveitis is characterized by inflammation primarily in the anterior chamber, according to the Standardization of Uveitis Nomenclature Working Group (SUN).[1] It is the most common type of uveitis, accounting for 50–90% of all uveitis diagnoses.[2, 3] Anterior uveitis can occur without any associated inflammation in other parts of the body or may be associated with systemic inflammatory disorders, including ankylosing spondylitis, sarcoidosis, interstitial nephritis, vasculitis, and inflammatory bowel diseases.[4] In particular, it is most commonly associated with a tissue type known as HLA-B27.[5]

Eyes with intraocular inflammation may show changes in choroidal thickness. For example, eyes with Behçet's disease show choroidal thickening during the active phase and a subsequent decrease in thickness after treatment.[6, 7] Choroidal thickness is correlated significantly with anterior and posterior ocular inflammation scores, and thickness change was associated with improved retinal vascular leakage.[6, 7] In addition, Vogt-Koyanagi-Harada disease is associated with choroidal thickening and after appropriate treatment, choroidal thickness can be reduced.[8, 9] These results show that choroidal thickness is associated with disease severity and activity.

Currently, there are a limited number of reports on choroidal thickness change in anterior uveitis. By definition, anterior uveitis is characterized by inflammation primarily in the anterior chamber. Therefore, this type of disease may not be associated with choroidal inflammation, and consequently one may speculate that no significant difference in choroidal thickness between eyes with and without anterior uveitis will exist. Géhl et al. showed that central choroidal thickness in anterior uveitis does not differ from that of normal controls.[10] Yan et al. demonstrated that choroidal thickness was reduced in patients with inactive anterior uveitis.[11] However, these studies are limited by healthy control groups, as controlling for several confounding factors including age, refractive errors, and diurnal variation[12–15] in the control group is technically challenging. Furthermore, only a limited number of subfoveal or perifoveal points were used to measure choroidal thickness in the studies and thus, point-by-point variations in the choroid and manual measurement may lead to inconsistent and biased results. Thickness mapping of the choroid may assess choroidal change more accurately by minimizing point-by-point variations.

In the present study, we assessed choroidal thickness in patients with unilateral, acute, anterior uveitis. We used automated measurements and choroidal thickness mapping to minimize bias and compared the thickness in eyes with active inflammation to that in the fellow eyes to match for factors affecting choroidal thickness. Furthermore, we measured choroidal thickness in both the peripapillary and macular areas and assessed the choroidal change in association with fluorescein angiography findings.

## Methods

### Patient selection

We retrospectively reviewed the medical records of 50 treatment-naïve, consecutive patients presenting with their first episode of unilateral anterior uveitis with an onset time of <1 week; patients were among 103 who visited the Department of Ophthalmology at Hanyang University Hospital between January 2014 and October 2016 and were diagnosed to have anterior uveitis associated with HLA-B27. This study was approved by Institutional Review Board of Hanyang University Hospital and was conducted in accordance with the Declaration of Helsinki. Our IRB waived the need for patients’ consent for their medical records to be used and the data were analyzed anonymously.

The diagnosis and grading of anterior uveitis was based on the Standardization of Uveitis Nomenclature (SUN) working group classification.[1] A comprehensive review of the patient's past medical history and diagnostic work-up was performed. The laboratory tests performed for the work-up included a complete blood count, erythrocyte sedimentation rate, C-reactive protein, an immune panel including antinuclear antibody and rheumatoid factor, HLA-B27 typing, specific viral serological assays for herpesviridae, venereal disease research laboratory test (VDRL), toxoplasmosis, toxocariasis, interferon-gamma release assay (QuantiFERON-TB Gold [Cellestis Limited, Carnegie, Australia]), and an angiotensin-converting enzyme test. Radiologic examinations such as chest x-rays and sacroiliac joint x-rays were also performed. With the exception of positive HLA-B27 results and/or abnormal sacroiliac radiographs, all laboratory and radiologic tests were negative, leading to the diagnosis of HLA-B27-associated anterior uveitis.

Exclusion criteria were as follows: (1) poor OCT images due to significant media opacity obscuring the choroidal layers (n = 3), (2) high myopia (myopia with a refractive error greater than 6.0 diopters, n = 2), and (3) combined macular and retinal diseases such as macular edema, central serous chorioretinopathy, age-related macular degeneration, and diabetic retinopathy (n = 1). Finally, 44 patients were included for analysis.

### Ophthalmic examinations and evaluations

All patients underwent comprehensive ophthalmic examinations including best-corrected visual acuities, slit-lamp examinations, intraocular pressure (IOP), refractive errors, and indirect ophthalmoscopy. Refractive error was measured with an autorefractometer (KW-1500, Kowa, Tokyo, Japan), and IOP was measured using non-contact tonometers (KT-500 automated tonometer, Kowa). Fluorescein angiography (FA) was performed at baseline using an F-10 confocal scanning laser ophthalmoscope (cSLO) (Nidek, Gmagori, Japan). Optical coherence tomography was performed in both eyes by swept-source optical coherence tomography (DRI-OCT, Topcon Inc., Tokyo, Japan) at baseline and after treating anterior chamber inflammation (Grade 0.5+ or less). Using a 1,050-nm wavelength light source, the device provides wide field three-dimensional (3D) macular volume scanning protocols, which consists of 256 B-scans, each comprised of 512 A-scans, covering a 12 mm × 9 mm area on the posterior pole, including the macular and peripapillary areas. All follow-up OCT images were obtained within 2 hours from the time of baseline OCT acquisition.

**Fig 1:**
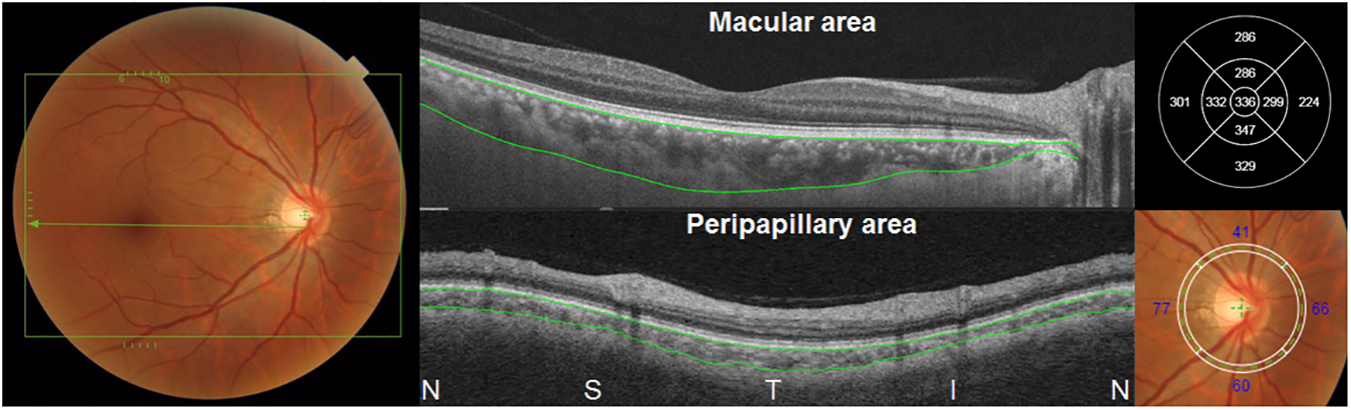
A photographic example of fundus photography and swept-source optical coherence tomography in an eye with acute anterior uveitis associated with human leukocyte antigen (HLA)-B27, covering a 12 (horizontal) × 9 (vertical)-mm area (box in left). Automated measurement of choroidal thickness by automated segmentation, which demarcates the outer border of the retinal pigment epithelium and the inner border of chorioscleral interface (indicated by lines), was performed using software provided by the manufacturer. A choroidal thickness map (upper right) was generated, and subfoveal choroidal thickness and parafoveal choroidal thicknesses were used for our analyses. Peripapillary thicknesses were also measured by automated segmentation and thickness mapping (lower), and the thicknesses on 4 quadrants (lower right) were used for our analyses. N = nasal; S = superior; T = temporal; I = inferior.

Central foveal thicknesses (CFT) in the 9 Early Treatment Diabetic Retinopathy Study (ETDRS) subfields were obtained by retinal thickness map analysis protocols. The choroidal thickness was automatically measured as the distance between the outer border of the RPE and the chorioscleral interface. Automated segmentation embedded in the software provided by the manufacturer determined the two borders, which were confirmed by two investigators (S.J.A. and J.H.K.). If there was any segmentation error, segmentation lines were manually corrected by the two investigators by consensus. In 9 patients, one or a few B-scans showed segmentation errors on the chorioscleral interface line, as shown in S1 Fig. The choroidal thickness map on the ETDRS grid was then obtained, as shown in Fig 1. Subfoveal choroidal thickness and nasal, superior, temporal, and inferior parafoveal choroidal thicknesses were utilized for our analyses. Peripapillary choroidal thicknesses were also automatically calculated using 3-mm diameter peripapillary circles centered on the optic disc (Fig 1, lower).

Late-phase FA images of the optic nerve head were obtained 4 min after dye injection and were analyzed to evaluate for the presence of optic disc leakage. Extravascular localization of dye on the optic disc was defined as optic disc leakage (S2 Fig).[16] For the final determination optic disc leakage, we compared the uveitic eye with the fellow eye, and if a greater degree of dye extravasation was observed in the uveitic eye, then the eye was judged to have optic disc leakage. Patients were divided into two subgroups based on the FA finding.

### Treatment

For the treatment of acute anterior uveitis, topical steroids (1% prednisolone acetate every one or two hours or four times a day depending on the severity) and cycloplegics (1% cyclopentolate or 1% atropine twice a day) were prescribed. For cases of severe inflammation (Grade 3+ or greater), a short course of oral corticosteroids (prednisolone 0.5 mg/kg/day initially with rapid tapering) was prescribed with or without a subtenon injection of triamcinolone acetonide (TRIAM, Shin Poong Pharm, Seoul, Korea). Patients were referred to the Department of Rheumatology at Hanyang University Hospital for further evaluation of spondyloarthropathy, and patients with ankylosing spondylitis received oral NSAIDs. A few patients were treated with tumor necrosis factor (TNF) inhibitors.

### Analyses

Descriptive statistics were used for demographic data and clinical characteristics, including FA findings. Choroidal thicknesses were compared between the uveitic eyes and the control (fellow) eyes by paired t tests. Thickness was compared between eyes with and without fluorescein findings using the Student’s t-test or Mann-Whitney test. The thicknesses were also compared before and after treatment using paired t-tests. In the 35 patients who had follow-up visits for equal to or longer than 1 month, the thicknesses were also compared between baseline and the time of inflammation control, using paired t tests.

Relative subfoveal choroidal thickening was defined as the subfoveal choroidal thickness of the eye with acute anterior uveitis minus that of the fellow eye. We tested for correlations between the relative subfoveal choroidal thickening and the degree of anterior chamber inflammation in order to evaluate for an association between choroidal thickening and the degree of intraocular inflammation.

Continuous data were presented as the mean ± standard deviation. Statistical analysis was performed using a statistical software package (SPSS, version 18.0; SPSS Inc., Chicago, IL, USA). P values less than 0.05 were considered statistically significant.

## Results

### Demographic and clinical characteristics

Table 1 shows the demographic data and clinical characteristics of the included patients. The mean patient age was 37.4 ± 11.4 years, ranging from 17 to 63 years, and 35 of 44 patients (79.5%) were male. The mean refractive error was -2.26 ± 1.79 and -2.31 ± 1.98 diopters in the uveitic and fellow eyes, respectively. Mean IOP at baseline was 13.6 ± 3.9 and 14.8 ± 3.7 mmHg in the uveitic and fellow eyes, respectively, but this difference was not statistically significant (P = 0.053 by paired t-test). The degree of anterior chamber inflammation was graded as follows: grade 0.5+ (n = 0), grade 1+ (n = 13), grade 2+ (n = 15), grade 3+ (n = 10), and grade 4+ (n = 6).

**Table 1:** Demographic data and clinical characteristics of included patients. Data are presented as number of patients (%) or mean ± standard deviation.

Characteristics	Numbers (%)
Age, yr	37.4 ± 11.4 (range: 17–63)
Sex, male	35 (79.5)
Laterality of uveitic eye, OD:OS	19: 25 (43.2:56.8)
Refractive errors, diopter	-2.26 ± 1.79 (range: -6-+1.0)
Mean IOP at baseline, mmHg	13.6 ± 3.9 (range: 4–23)
Inflammation grade (SUN classification), Grade 0.5+:1+:2+:3+:4+	0:13:15:10:6 (0:29.5:34.1:22.7:13.6)
Time of OCT image acquisition	11:42 AM (range: 9:01 AM-14:12 PM)
Central foveal retinal thickness, μm	239.5 ± 22.5 (range: 179–290)

IOP = intraocular pressure; OCT = optical coherence tomography; SUN = the Standardization of *Uveitis* Nomenclature working group

### Choroidal thickness in eyes with anterior uveitis and the fellow eyes

**Fig 2:**
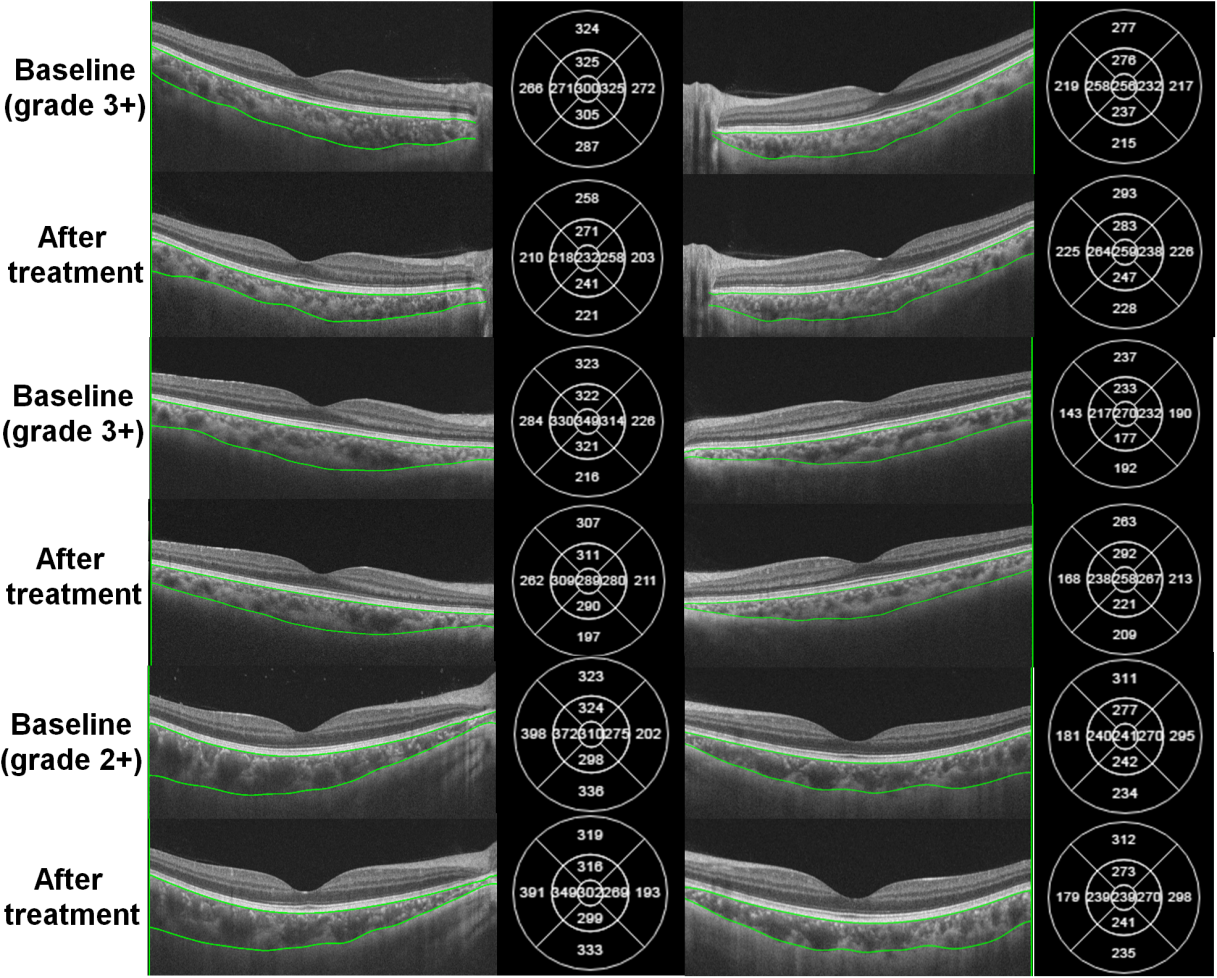
Swept-source optical coherence tomography (SS-OCT) B-scan images in three representative patients with unilateral, acute, anterior uveitis. Compared to the fellow eyes (right), the eyes with acute anterior uveitis (left) show choroidal thickening of the macular area, which can be also determined by numerical values of choroidal thicknesses of the thickness maps (right side of each figure). Choroidal thickness decreased after topical and/or systemic corticosteroid therapy in the eyes with uveitis. In the fellow eyes, however, there were no significant changes in choroidal thickness before and after the treatment. The numbers within parentheses indicate the grades of anterior chamber inflammation.

**Fig 3:**
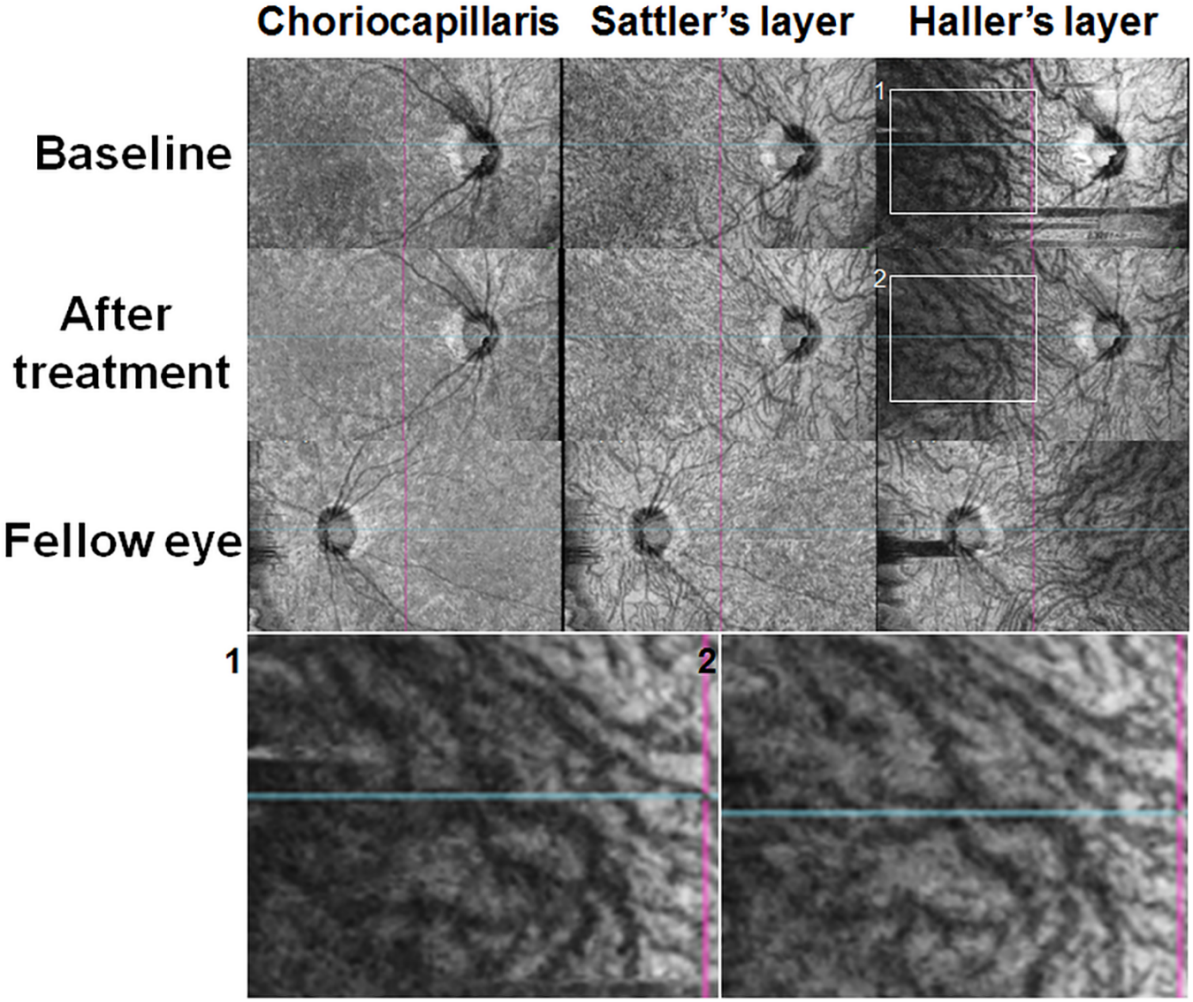
En face choroidal imaging in an eye with acute anterior uveitis and the fellow eye. The choriocapillaris or Sattler’s layer shows no remarkable change between uveitic and fellow eyes or before and after the treatment in the uveitic eye. However, the Haller’s layer in the eye with active uveitis shows dilation of the large choroidal vessels on the macular area at baseline (Box 1) compared to images obtained after treatment (Box 2) or fellow eye, which is more remarkable in magnified images on the macular area (lower).

Fig 2 demonstrates OCT B-scan images in three randomly selected patients with unilateral, acute, anterior uveitis. There were no remarkable morphological abnormalities observed on the uveitic eye. However, the choroid in the uveitic eyes seemed thickened compared to the fellow eyes. For example, choroidal thickness maps in the patient with grade 3+ inflammation (top) indicated that subfoveal choroidal thicknesses in the uveitic eye and the fellow eye were 300 and 256 μm, respectively. En face images of the choroid in a randomly selected patient (Fig 3) show dilated large choroidal vessels (right column) particularly on the macular area (indicated by boxes) at baseline, compared to the image obtained after treatment and that of the fellow eye at baseline, which become more remarkable in magnified images of the macular area (lower).

**Fig 4:**
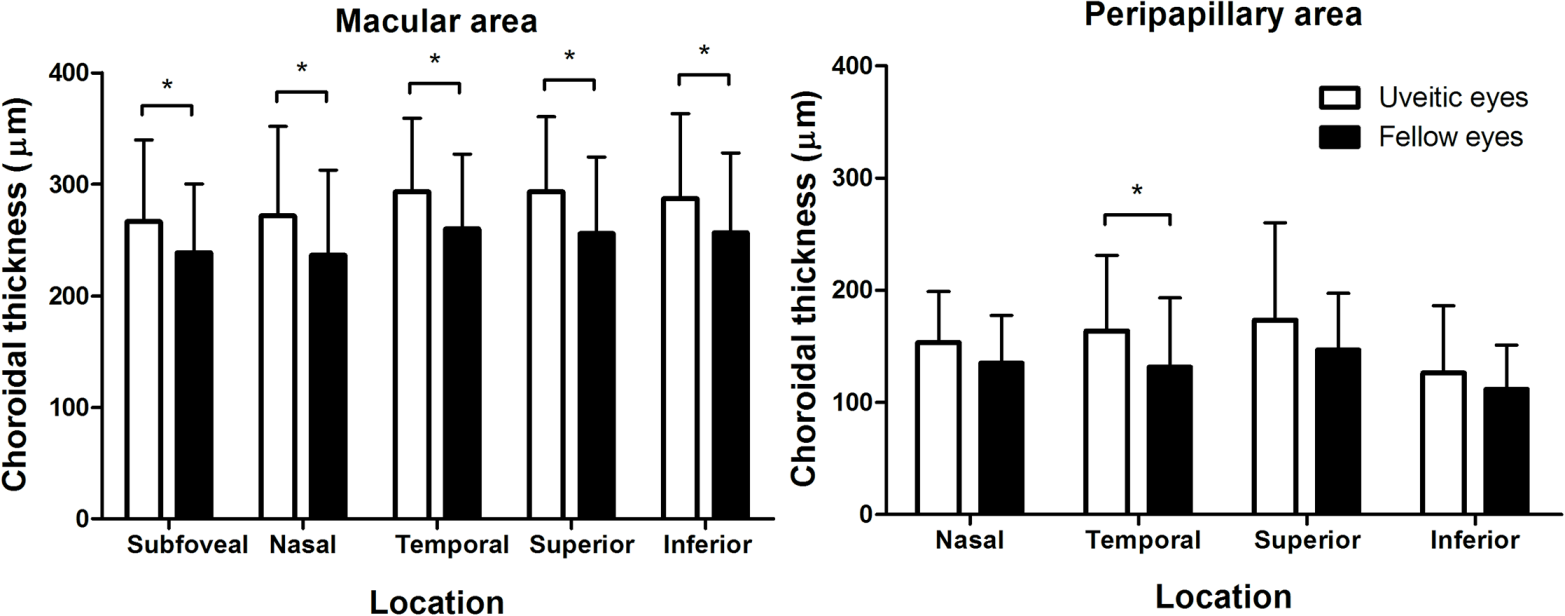
Comparison of macular and peripapillary choroidal thicknesses between eyes with acute anterior uveitis and the fellow eyes. There are significant differences in subfoveal and parafoveal choroidal thicknesses between the uveitic and fellow eyes. Asterisk (*) indicates statistical significance (P <0.05).

Fig 4 compares macular choroidal thicknesses between the uveitic and fellow eyes in the included patients. There was a significant difference in subfoveal choroidal thickness (266.6 ± 73.4 and 238.7 ± 61.5 μm in uveitic and fellow eyes, respectively, P<0.001 by paired t-test) and all 4 parafoveal thickness (all P <0.05). Peripapillary choroidal thickness did not vary significantly between the two groups (P >0.05), except in temporal quadrant (P = 0.009). S1 Table compares the clinical characteristics between the uveitic and fellow eyes. There were no significant differences in refractive errors, IOP at baseline, and CFT (all P >0.05)

There was no significant correlation between the degree of anterior uveitis and subfoveal choroidal thickness (r = 0.235; P = 0.124). However, correlation analyses between relative subfoveal choroidal thickening and the degree of anterior chamber inflammation revealed a significant correlation (correlation coefficient [r] = 0.341, P = 0.023), indicating that eyes with more severe anterior chamber inflammation showed greater choroidal thickening compared to fellow eyes.

### Choroidal thickness in subgroups separated based on fluorescein angiography findings

Based on the presence of optic disc leakage on FA, 44 patients were divided into ‘with disc leakage’ (n = 23) and ‘without disc leakage’ groups (n = 21). S2 Table shows a comparison of clinical characteristics between the two groups, and reveals no significant difference with respect to refractive errors and IOP. Macular choroidal thicknesses were negligibly different between the two groups, as shown in S3 Fig. Peripapillary choroid tended to be thicker in eyes with optic disc leakage than in those without; however, this was not statistically significant (all P >0.05).

### Choroidal thickness before and after treatment in anterior uveitis

**Fig 5:**
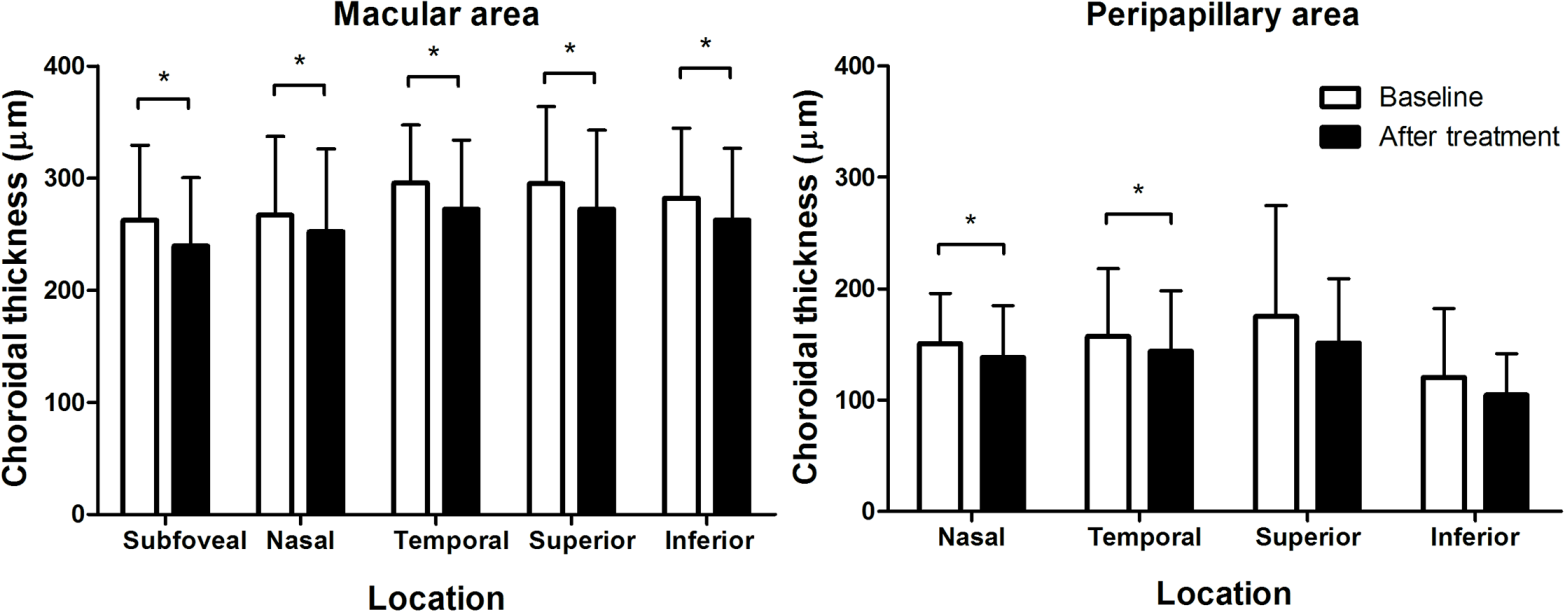
Comparison of macular and peripapillary choroidal thicknesses before and after treatment of anterior uveitis. Subfoveal and parafoveal choroidal thickness thinned significantly following treatment (all P <0.05). Peripapillary choroidal thicknesses show significant difference only in nasal and temporal quadrants. Asterisk (*) indicates statistical significance (P <0.05).

After administration of topical steroids with or without systemic/periocular corticosteroids, the anterior chamber inflammation was resolved to be grade 0.5+ or absent according to SUN classification in all the patients. Time from initiation of treatment to resolution of inflammation (and to post-treatment OCT imaging) ranged from 1 to 4 weeks (mean, 2.1 weeks), depending on the severity of baseline anterior chamber inflammation, whereas the duration of therapy ranged from 2 to 12 weeks on the average of 5.3 ± 2.5 weeks. There was no significant difference in mean IOP of the uveitic eye before and after treatment (13.8 ± 3.9 and 14.3 ± 2.9 before and after treatment, respectively, P = 0.372 by paired t-test). The OCT image taken after the resolution of anterior uveitis showed choroidal thinning when compared to the baseline image, as shown in Fig 2, although the fellow eye showed no significant change between the two images. Overall, subfoveal choroidal thickness changed significantly from 262.5 ± 66.9 to 239.5 ± 61.0 μm (Fig 5, P<0.001 by paired t-test). Parafoveal choroidal thickness significantly decreased in all 4 quadrants (S3 Table, all P <0.05); however, there was no significant difference in superior or inferior peripapillary choroidal thickness (Fig 5B) before and after treatment (both P >0.05) whereas the nasal and temporal choroid showed significant differences in thickness following treatment (P = 0.010 and 0.027 for nasal and temporal choroid, respectively).

## Discussion

The present study provides a detailed assessment of choroidal thickness in peripapillary and macular locations using SS-OCT, which enables better imaging of deeper structures, like the chorioscleral interface, than conventional spectral domain OCT. Using automated measurements, our study showed choroidal thickening on the macular area in eyes with acute anterior uveitis and a reduction in thickness after treatment. A dose-response relationship between relative choroidal thickening and the degree of anterior chamber inflammation further strengthen the association between anterior uveitis and choroidal thickening. Our study suggests that inflammation may be affecting the eye more widely than clinicians observe from biomicroscopic examinations.

Previous studies demonstrated that in several diseases showing intraocular inflammation, the choroid thickens at times of active acute inflammation and thins during the chronic phase.[17, 18] The conditions associated with choroidal thickening include Vogt-Koyanagi-Harada disease, sympathetic ophthalmia, Behçet’s disease, and toxoplasma retinochoroiditis.[17, 18] These conditions commonly involve the posterior segment, and choroidal inflammation is considered to be associated with choroidal thickening. Our results indicate that subclinical or latent choroidal inflammation may also be present in active anterior uveitis, although the choroid is anatomically disconnected from the anterior segment. The choroidal changes observed in HLA-B27-associated acute anterior uveitis may also occur in other types of acute anterior uveitis (S4 Fig), as our pilot study on idiopathic anterior uveitis also showed a significant difference in subfoveal choroidal thickness between uveitic and fellow eyes (230.3 ± 86.0 vs. 200.3 ± 60.7 μm, P = 0.024). Our results suggest that choroidal evaluation may be useful for monitoring disease activity, assessing combined choroidal inflammation, and making treatment decisions in anterior uveitis.

Rodrigues et al. reported that 17.4% of eyes with HLA-B27-associated uveitis showed posterior segment involvement.[19] They reported several manifestations such as severe and diffuse vitritis and cystoid macular edema for the posterior segment involvement and emphasized that the posterior involvement is an under-recognized phenomenon in HLA-B27-associated uveitis. Although our cases were classified into anterior uveitis according to SUN classification as (1) the primary site of inflammation was the anterior chamber and (2) vitritis was absent or mild, the choroid may also be involved in our cases as posterior segment involvement of HLA-B27-associated uveitis. For HLA-B27-associated uveitis combined with remarkable choroidal thickening, our results might support the use of systemic corticosteroid therapy in addition to topical anti-inflammatory treatment for anterior uveitis, as the topical therapy may not be sufficient for treating choroidal inflammation.

The causes of choroidal thickening in the acute phase of intraocular inflammation may be (1) inflammatory infiltration leading to increased choroid volume and (2) increased blood perfusion in the choroid, which is possibly associated with the inflammatory process. We propose that the choroidal thickening in our patients may be associated with increased blood flow, as (1) there was no definite change in the choroidal morphology on OCT B-scans (Fig 2) and (2) en face images of the uveitic eyes showed dilation of large choroidal vessels on the macular area (Fig 3). Unfortunately, current SS-OCT devices are unable to obtain data on blood flow, which is necessary to confirm our hypothesis. However, en face choroidal imaging using SS-OCT provided depth-resolved images of medium- to large-sized choroidal vasculatures and thus facilitated identification of the vascular changes in the choroid, which is challenging to obtain from conventional B-scan images. Our study also suggests that this imaging modality can be applied to other types of uveitis more extensively, to demonstrate choroidal changes in the inflammatory disorders. This study also assessed optic disc leakage in patients with acute anterior uveitis. In normal eyes, the optic nerve head can have some fluorescein leakage due to the normally leaking peripapillary choriocapillaris.[20] About half of the patients showed greater leakage in the optic disc, while others showed symmetric leakage or staining of the optic disc, indicating that the eyes with acute anterior uveitis may have greater leakage from the peripapillary choriocapillaris. This might provide FA evidence for choroidal inflammation in eyes with active anterior uveitis and may also explain the observed trend toward peripapillary choroidal thickening in eyes with disc leakage, albeit statistically nonsignificant.

This study showed a significant decrease in choroidal thickness in eyes with active anterior uveitis after treatment. The result is consistent with previous studies showing that several diseases have increased choroidal thickness in the acute phase and reduced thickness following corticosteroid treatment.[6, 8] Kola et al. showed subfoveal choroidal thickening in patients with ankylosing spondylitis.[21] Among the patients included in this study, 32 (72.7%) had ankylosing spondylitis; however, there were no significant differences in choroidal thicknesses between those with and without ankylosing spondylitis at baseline (P > 0.05). Furthermore, those with and without ankylosing spondylitis showed similar choroidal responses (thinning) following treatment for anterior uveitis. Therefore, choroidal thickening in eyes with HLA-B27-associated anterior uveitis might be associated with anterior uveitis, rather than ankylosing spondylitis. Yan et al. showed reduced choroidal thickness in patients with inactive anterior uveitis, which seems to conflict with our results.[11] However, the authors showed an inverse correlation between disease duration and choroidal thickness in anterior uveitis. As the number of recurrences and the duration may be important confounding factors affecting choroidal thickness, we included patients with a first presentation of acute anterior uveitis to minimize the effect of such confounding factors. Thus, choroidal thickening in our patients may not conflict with previous results. The activity of anterior chamber inflammation and the chronicity of the condition should be carefully evaluated, as they both seem to affect choroidal thickness.

Several limitations of the present study require careful interpretation. First, we studied a relatively small sample size, which may not be representative of all patients with active anterior uveitis. Furthermore, although HLA-B27 is one of the most common causes of anterior uveitis,[5] this type may not represent the whole population of anterior uveitis patients. Additionally, the retrospective design of this study may have an intrinsic drawback with respect to bias. Furthermore, although there was an insignificant difference in the mean time of OCT acquisition before and after treatment and although the follow-up OCT images were obtained within 2 hours from the OCT acquisition time at baseline, diurnal variation may have affected our results. Despite these limitations, our study used wide volume SS-OCT scans to analyze extensive areas of the choroid including peripapillary and macular areas. This study also minimized potential bias on the choroidal thickness measurement, present in previous studies using manual measurement, by automated measurement and choroidal thickness mapping.

In conclusion, this study showed subfoveal choroidal thickening in eyes presenting with the first episode of acute anterior uveitis and its thinning following anti-inflammatory treatment. These findings indicate subclinical choroidal inflammation and choroidal evaluation in the disease may provide information on disease activity. Further studies with a larger sample size are warranted to validate the usefulness of choroidal evaluation in these diseases.

## Supporting information

S1 Fig Optical coherence tomography (OCT) images in a representative eye showing segmentation errors made by automated segmentation (middle). Manual segmentation (bottom) was performed to correct the error.
(TIF)

S2 Fig Fluorescein angiography images of both eyes in a patient with unilateral acute anterior uveitis obtained 4 minutes after dye injection. Optic disc leakage was determined when (1) extravascular localization of the dye on the optic disc was present in the uveitic eye (left) and (2) when the degree of dye leakage was more severe in the eye (left) than in the fellow eye (right).
(TIF)

S3 Fig Comparison of macular and peripapillary choroidal thicknesses between eyes with and without optic disc leakage. There were no significant differences between the two groups, although peripapillary choroidal thicknesses in eyes with optic disc leakage showed a thickening trend in all the 4 quadrants.
(TIF)

S4 Fig Optical coherence tomography images in a patient having HLA-B27-associated anterior uveitis with an onset time of 15 days (left) and those in a patient with acute idiopathic anterior uveitis (right). (TIF)

S1 Table Comparison of clinical characteristics between eyes with acute anterior uveitis and the fellow eyes. Data are presented as mean ± standard deviation.
(DOCX)

S2 Table Comparison of clinical characteristics between eyes with and without optic disc leakage. Data are presented as number of patients (%) or mean ± standard deviation.
(DOCX)

S3 Table Mean choroidal thickness (± standard deviation) in eyes with uveitis and the fellow eyes before and after the treatment. (DOCX)

S1 Dataset Subfoveal choroidal thickness data in the uveitic and fellow eyes. (XLSX)
