# Supplementary figures and images for: Choroidal change in acute anterior uveitis associated with human leukocyte antigen-B27
Source: PLoS One. 2017 Jun 28;12(6):e0180109. doi: 10.1371/journal.pone.0180109 (PMC5489203; doi:10.1371/journal.pone.0180109)

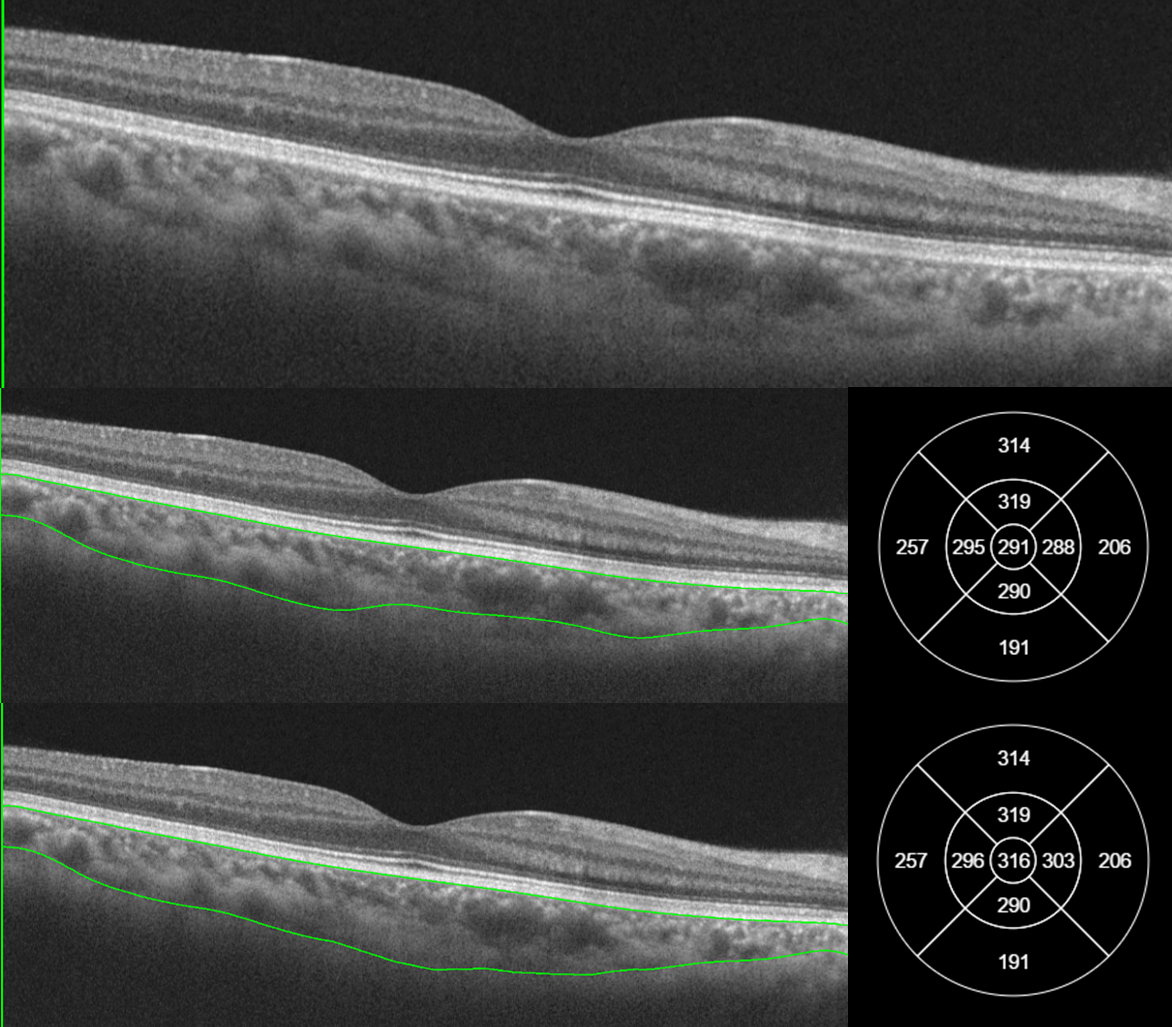

Supplement: S1 Fig — Manual segmentation (bottom) was performed to correct the error. (TIF) [file pone.0180109.s001.tif]

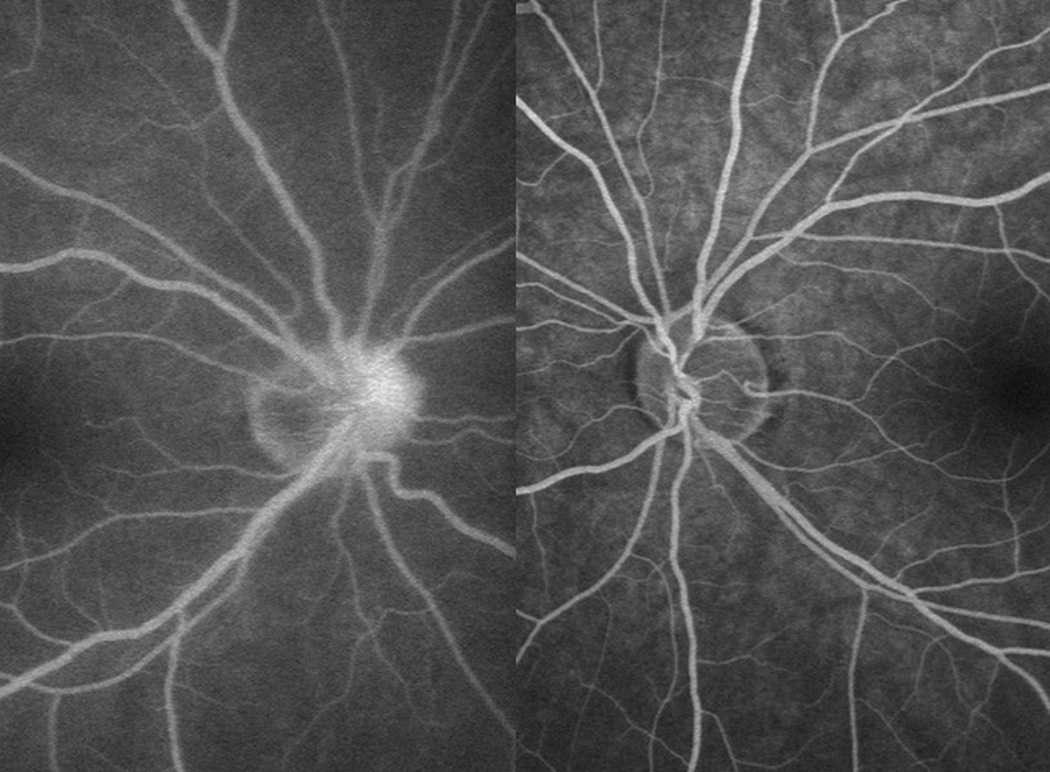

Supplement: S2 Fig — Optic disc leakage was determined when (1) extravascular localization of the dye on the optic disc was present in the uveitic eye (left) and (2) when the degree of dye leakage was more severe in the eye (left) than in the fellow eye (right). (TIF) [file pone.0180109.s002.tif]

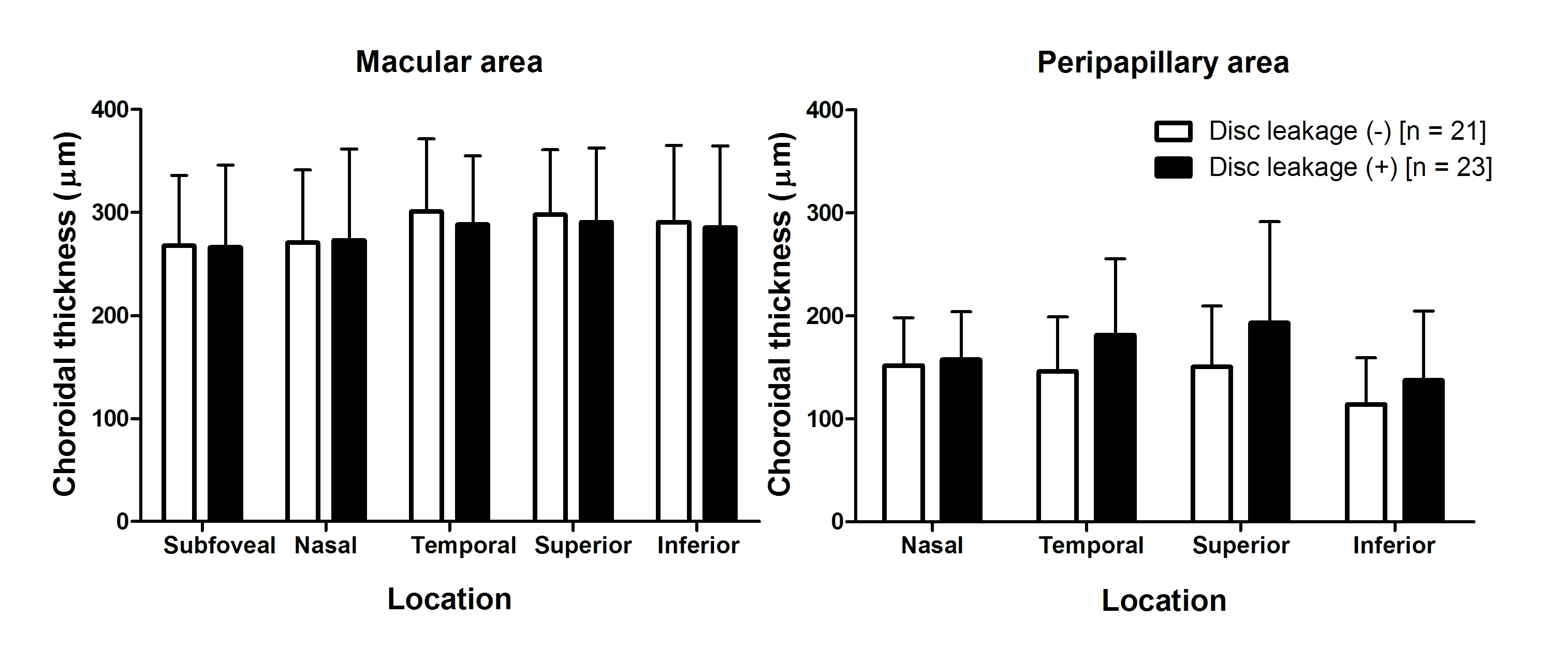

Supplement: S3 Fig — There were no significant differences between the two groups, although peripapillary choroidal thicknesses in eyes with optic disc leakage showed a thickening trend in all the 4 quadrants. (TIF) [file pone.0180109.s003.tif]

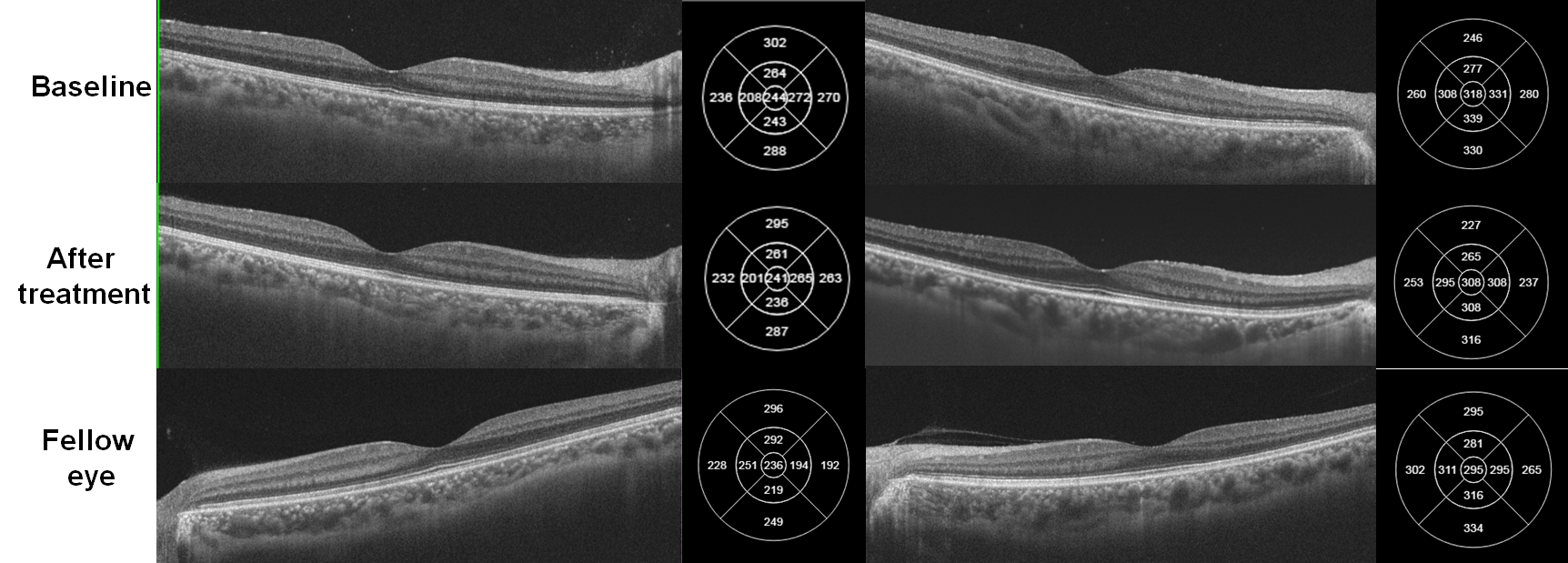

Supplement: S4 Fig — (TIF) [file pone.0180109.s004.tif]
