# Supplementary material for: Choroidal change in acute anterior uveitis associated with human leukocyte antigen-B27
Source: PLoS One. 2017 Jun 28;12(6):e0180109. doi: 10.1371/journal.pone.0180109 (PMC5489203; doi:10.1371/journal.pone.0180109)
Supplement: S1 Table — Data are presented as mean ± standard deviation. (DOCX) [file pone.0180109.s005.docx]

**S1 Table. Comparison of clinical characteristics between eyes with acute anterior uveitis and the fellow eyes.** Data are presented as mean ± standard deviation.

| **Characteristics** | **Uveitic eyes** | **Fellow eyes** | **P value** |
| --- | --- | --- | --- |
| Refractive errors, diopter | -2.26 ± 1.79 | -2.31 ± 1.98 | 0.627 |
| Mean IOP at baseline, mmHg | 13.6 ± 3.9 | 14.8 ± 3.7 | 0.053 |
| Mean time of OCT acquisition | 11:42 AM | 11:41 AM | N/A |
| Central foveal retinal thickness, μm | 239.5 ± 22.5 | 235.5 ± 22.7 | 0.060 |

IOP=intraocular pressure; N/A=not applicable; OCT=optical coherence tomography
