# Supplementary material for: Choroidal change in acute anterior uveitis associated with human leukocyte antigen-B27
Source: PLoS One. 2017 Jun 28;12(6):e0180109. doi: 10.1371/journal.pone.0180109 (PMC5489203; doi:10.1371/journal.pone.0180109)
Supplement: S2 Table — Data are presented as number of patients (%) or mean ± standard deviation. (DOCX) [file pone.0180109.s006.docx]

**S2 Table. Comparison of clinical characteristics between eyes with and without optic disc leakage.** Data are presented as number of patients (%) or mean ± standard deviation.

| **Characteristics** | **With disc leakage (n=23)** | **Without disc leakage (n=21)** | **P value** |
| --- | --- | --- | --- |
| Age, yrs | 38.7 ± 10.3 | 35.9 ± 12.5 | 0.423 |
| Sex, male (%) | 16 (69.6) | 19 (90.5) | 0.137 |
| Refractive errors, diopter | -2.28 ± 1.82 | -2.23 1.77 | 0.918 |
| Mean IOP at baseline, mmHg | 13.0 ± 4.0 | 14.2 ± 3.8 | 0.345 |
| Inflammation grade (SUN classification) | 2.1 ± 1.1 | 2.3 ± 0.9 | 0.621 |
| Mean time of OCT acquisition | 11:51 AM | 11:33 AM | N/A |
| Central foveal retinal thickness, μm | 243.0 ± 18.2 | 235.6 ± 26.3 | 0.282 |

IOP=intraocular pressure; N/A=not applicable; OCT=optical coherence tomography; SUN=the Standardization of Uveitis Nomenclature working group
