# Supplementary material for: Choroidal change in acute anterior uveitis associated with human leukocyte antigen-B27
Source: PLoS One. 2017 Jun 28;12(6):e0180109. doi: 10.1371/journal.pone.0180109 (PMC5489203; doi:10.1371/journal.pone.0180109)
Supplement: S3 Table — (DOCX) [file pone.0180109.s007.docx]

**S3 Table**. Mean choroidal thickness (± standard deviation) in eyes with uveitis and the fellow eyes before and after the treatment.

|  | **Before treatment** | **After treatment** |
| --- | --- | --- |
| **Uveitic eye**  Central foveal  Parafoveal nasal  Parafoveal temporal  Parafoveal superior  Parafoveal inferior | 266.6 ± 73.4  271.9 ± 80.0  293.4 ± 65.8  293.5 ± 66.9  287.4 ± 75.8 | 239.5 ± 61.0  252.2 ± 74.1  272.4 ± 61.7  272.2 ± 70.6  262.6 ± 64.1 |
| **Fellow eye**  Central foveal  Parafoveal nasal  Parafoveal temporal  Parafoveal superior  Parafoveal inferior | 238.7 ± 61.5  236.5 ± 76.5  260.0 ± 67.2  256.1 ± 68.5  256.8 ± 71.4 | 250.5 ± 73.6  234.3 ± 73.9  254.3 ± 59.4  265.4 ± 74.3  243.8 ± 61.0 |
